# Sequencing and characterization of mitochondrial genome of *Fusarium* sp. (Hypocreales: Nectriaceae)

**DOI:** 10.1080/23802359.2017.1365653

**Published:** 2017-08-16

**Authors:** Qiang Li, Guiqi Bi, Daihua Lu, Cheng Chen

**Affiliations:** a Institute of plant protection, Sichuan Academy of Agricultural Sciences, Chengdu, China;; b College of Marine Life Sciences, Ocean University of China, Qingdao, China

**Keywords:** Fusarium, mitogenome, phylogenetic analysis

## Abstract

In this study, we presented the complete mitochondrial genome of *Fusarium* sp. It has a total length of 47, 74 bp, and the base composition of the mitogenome is as follows: A (34.1%), T (33.5%), C (14.7%), and G (17.7%). The mitogenome contains 23 protein-coding genes, 1 ribosomal RNA (rRNA), and 26 transfer RNA (tRNA) genes, all coded on the same strand of DNA. The gene order is identical to that of the other *Fusarium* mitogenomes. The taxonomic status of the *Fusarium* sp. mitogenome exhibits a closest relationship with *F. oxysporum*, but varied in the structure of mitochondrial genome.

*Fusarium* is a large genus of filamentous fungi, which is widely distributed in soil and associated with plants (Nelson et al. 1994). Most species in this genus are harmless saprobes and also include a number of economically important plant pathogenic species (Moretti 2009). *F. incarnatum*–*F. equiseti* species complex was reported causes stipe rot disease on *Morchella importuna* in China (Guo et al. 2016). Diseases caused by *Fusarium* spp. have caused great losses to crop growers. The taxonomy of the genus is complex. A number of different schemes have been used, and up to 1000 species have been identified at times (Watanabe et al. 2011; Summerell et al. 2010). In the present study, the complete mitogenome sequence of *Fusarium* sp. has been determined, which provides the reference sequence of mitogenome for *Fusarium* sp. that may be utilized for the determination of population evolution and phylogeny in the future.

The specimen (*Fusarium* sp.) was isolated from the soil of diseased *Morchella sextelata* in Pujiang, Sichuan, China (103.29E; 30.20N) and was stored in Sichuan Academy of Agricultural Sciences (No. SAAS1). The total genomic DNA of obtained mycelia was extracted using Fungal DNA Kit D3390-00 (Omega Bio-Tek, Norcross, GA) according to the manufacturer’s instructions and was stored in the sequencing company (BGI Tech, Shenzhen, China). Purified DNA was used to construct the sequencing libraries following the instructions of NEBNext^®^ Ultra™ II DNA Library Prep Kit (NEB, Beijing, China). Whole genome sequencing was performed using the Illumina HiSeq 2500 Platform (Illumina, San Diego, CA). Multiple steps were used for quality control and *de novo* assembly of the mitogenome according to Bi (2017). Adapters and low-quality reads were removed using the NGS QC Toolkit (Patel and Jain 2012). The obtained clean reads were screened out by Bowtie 2 (Langmead and Salzberg 2012) using other *Fusarium* mitochondrial genomes as references, and then assembly as implemented by SPAdes 3.9.0 (Bankevich et al. 2012). Gaps among contigs were filled by using MITObim V1.9 (Hahn et al. 2013). The determined genome was annotated using the MFannot tool (http://megasun.bch.umontreal.ca/cgi-bin/mfannot/mfannotInterface.pl), combined with manual corrections. tRNAs were annotated by ARWEN web server (Laslett and Canbäck 2008).

The total length of *Fusarium* sp. circular mitogenome is 47,874 bp, which showed high similarity with *Fusarium* species both in size, structure, and gene content. This mitogenome was submitted to GenBank database under accession no. MF538632. The circular mitogenome contains 23 protein-coding genes, 1 ribosomal RNA (rRNA), and 26 transfer RNA (tRNA) genes (Figure 1). The base composition of the genome is as follows: A (34.1%), T (33.5%), C (14.7%), and G (17.7%), all coded on the same strand of DNA.

**Figure 1.**
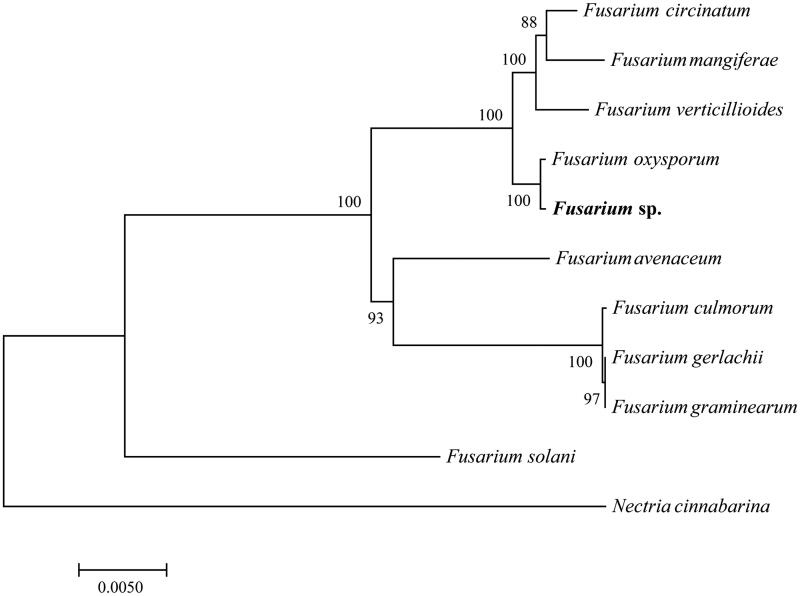
Phylogenetic relationships among 10 *Fusarium* mt genomes. This tree was drawn with *Nectria cinnabarina* as an out-group. All nodes exhibit above 90% posterior probability (PP) and 85% RAxML supported bootstraps. The length of branch represents the divergence distance. Mitogenome accession numbers used in this phylogeny analysis are as follows: *F. circinatum* (JX910419), *F. mangiferae* (KP742838), *F. verticillioides* (JN041210), *F. oxysporum* (KR952337), *F. avenaceum* (JQGE01000000), *F. culmorum* (KP827647), *F. gerlachii* (KM486533), *F. graminearum* (HG970331), *F. solani* (JN041209), and *Nectria cinnabarina* (KT731105).

To validate the phylogenetic position of *Fusarium* sp., the genome-wide alignment of 10 *Fusarium* mitogenomes was constructed by HomBlocks (https://github.com/fenghen360/HomBlocks) (Bi 2017). Bayesian analysis (BI) and maximum likelihood (ML) were used to construct the phylogenetic trees with all protein-coding genes and rRNA according to Bi (2017). Bootstrap values were calculated using 1000 replicates to assess node support. As shown in the phylogenetic tree (Figure 1), the taxonomic status of the *Fusarium* sp. based on mitogenome exhibits a closest relationship with *F. oxysporum* (GenBank accession number: KR952337).
